# Gender-affirming care through the lens of abnormal illness behaviour and abnormal treatment behaviour

**DOI:** 10.1177/10398562241276978

**Published:** 2024-08-29

**Authors:** Patrick Clarke, Andrew J Amos

**Affiliations:** Faculty of Health and Medical Science, Adelaide Health and Medical Sciences building, University of Adelaide, North Adelaide, SA, Australia; Division of Tropical Health and Medicine, College of Medicine and Dentistry, 104397 James Cook University, Townsville, QLD, Australia

**Keywords:** abnormal illness behaviour, abnormal treatment behaviour, child and adolescent psychiatry, psychoanalysis, psychotherapy

## Abstract

**Objective:**

To describe the increasing number and changing demographics of patients presenting with gender dysphoria and provide an account of patient- and clinician-related factors which may have contributed to these changes. The concept of abnormal illness behaviours introduced by Pilowsky, and its extension to the concept of abnormal treatment behaviours by Singh, provides a framework for understanding healthy and pathological interactions between gender dysphoria patients and their doctors.

**Conclusions:**

Abnormal illness behaviours driven by the reinforcing contingencies of gender-affirming care may explain, in part, the increasing number and changing demographics of gender dysphoria, as well as the increasing incidence of desistance and detransition. The under-diagnosis and under-treatment of mental health disorders by clinicians treating these patients are examples of abnormal treatment behaviours. Uncritical affirmation of patient reported gender identity appears likely to conceal unconscious motivations of some patients and clinicians, increasing the risks of harm to both.

Over the past 10 years, there has been a dramatic increase in the number of people presenting with gender dysphoria or gender diversity seeking gender transition (see Table 1 for definitions).^1^ Previously, most people seeking transition were natal males who began identifying as females pre-pubertally or in middle age. The increase in overall presentations is largely due to growing numbers of natal females who began identifying as males in adolescence, which was previously rare and has been associated with social contagion.^2^

**Table 1.** Definitions in gender medicine

***Conversion therapy*** : Conversion therapy refers to a ‘treatment or other practice the purpose, or purported purpose, of which is to change or suppress a person’s sexual orientation or gender identity’
***Desistance***: The reversion of a gender identity in conflict with biological sex to a gender identity consistent with biological sex occurring before the initiation of any affirming medical or surgical treatments
***Detransition***: The reversion of a gender identity in conflict with biological sex to a gender identity consistent with biological sex occurring after the initiation of any affirming medical or surgical treatments
***Diagnostic overshadowing***: A mechanism in which the diagnosis of one condition (such as gender dysphoria) makes it less likely that differential diagnoses or comorbid conditions will be recognised when they do in fact exist
***Gender* :** Gender refers to the state of being male, female, or other, and/or masculine, feminine, and other, with regard to personal, social, and cultural characteristics, rather than genetic, hormonal, or anatomical characteristics
** *Gender affirmation* **: Any social, medical, surgical, or other intervention intended to reinforce the experience of a patient’s chosen gender identity instead of a gender identity consistent with their biological sex
***Gender-affirming interventions***: Any medical or allied health intervention provided by a gender service with the intention of affirming a self-reported gender identity. Typically divided into the three categories of social, medical, and surgical interventions. Technically the purpose of puberty blockers is to delay the onset of puberty to facilitate assessment and initiation of definitive gender-affirming treatment rather than necessarily affirming gender identity in itself
***Gender-affirming model of care (GAMOC)* :** A specification of the assumed knowledge, clinical practices, organisational structures, personnel, and resources required to achieve a minimum standard of care for gender diverse patients treated by a specific health service using gender-affirming care
***Gender diversity***: Used both to describe the broad group of people with a gender identity that is in conflict with their biological sex, including specific categories such as transgender and non-binary; and to describe the position that gender identities in conflict with biological sex are part of the normal variation of human development
***Gender dysphoria (GD – DSM-V)* :** Gender dysphoria is defined in the Diagnostic and Statistical Manual of Mental Disorders 5th edition Text Revised (DSM-5-TR) as a ‘marked incongruence between one’s experienced or expressed gender and one’s assigned gender, associated with clinically significant distress or impairment in functioning’
***Gender identity (GID)* :** Gender identity refers to an individual’s personal, internal sense of being, in relation to gender, and their expression of that sense of being. Gender identity is distinct from chromosomal sex and anatomical sex. Some people report a gender identity which is incongruent with their biological sex
***Gender incongruence (GI – ICD-11)* :** Gender incongruence is defined in the International Classification of Diseases 11th revision (ICD-11), as a ‘marked and persistent incongruence between a person’s experienced gender and assigned sex’
***Gender service***: Name used to describe the specialised clinical units established to provide the gender-affirming model of care to gender diverse patients in Australia’s public health services
***Gender transition***: A clinical or non-clinical process which results in a patient changing their gender identity from one category to another. Generally not understood to include detransition
***Hormone therapy***: Hormonal treatments administered with the intention of making patients’ physical and social experiences more consistent with their gender identity
***Medical affirmation***: Medical treatment intended to modify a patient’s appearance, physical characteristics, or emotional experiences in order to be more consistent with their gender identity
***Minority stress***: Theory that explains the distress experienced by minority groups such as LGBT people and ethnic minorities as the result of external processes like stigmatisation and discrimination rather than internal processes such as mental illness
***Natal male/female***: Sex present or assigned at birth
***Non-binary***: Nonspecific term encompassing all people who experience a gender identity that is not defined in terms of the two categories male and female
***Puberty blockers***: In the GAMOC refers to medications prescribed to adolescents with the intention of delaying the onset and progression of puberty to reduce distress, facilitate assessment, or plan definitive treatment
***Regret***: Broadly defined as a negative, cognitive-based emotion involving counterfactual inference and feelings of personal agency or self-blame with respect to partial or complete gender transition
***Sex* :** Sex refers to the set of biological characteristics such as genes that define humans as female or male. While these sets of biological characteristics are not mutually exclusive, as there are individuals who possess features of both male and female sex, they differentiate humans as males and females in the vast majority of people
***Social affirmation***: The creation of a social environment consistent with the patient’s gender identity and not their biological sex
***Social contagion***: Theory that some presentations of gender diversity are caused by social mechanisms such as modelling rather than innate developmental processes, based on reports in which a number of people from the same friendship networks seek to transition close in time despite limited history of reported gender diverse experiences
***Surgical affirmation***: Surgical interventions intended to modify a patient’s appearance, physical characteristics, or emotional experiences to be more consistent with their gender identity
***Transgender***: Describes a person with a gender identity that is the opposite to their natal sex

These changes accompanied the growing influence of the gender-affirming model of care (GAMOC) on Australian and New Zealand gender services. The GAMOC was pioneered in the Netherlands^3^ and is promoted by an international network of groups including the Australian Professional Association for Trans Health (AusPATH)^4^ and the World PATH (WPATH).^5^ The GAMOC assumes that all people have a gender identity that is not determined by biological sex; assumes that patients are unquestionable experts about their gender identity, regardless of age; and recommends that professionals, family, and community always affirm patient reported gender identity.

Alongside puberty blockers intended to increase the time available to make decisions about the management of gender dysphoria, the GAMOC includes three modes of intervention affirming gender identity by social, medical, or surgical means. The most ambitious claim presented by GAMOC advocates in support of affirming interventions is that they are potentially lifesaving, citing the increased risk of suicide in gender diverse patients, which they associate with minority stress and societal prejudice.^4,5^ However, methodological limitations in this literature prevent the conclusion that GAMOC has a causal role in reducing suicide.^6–11^

In addition, a NICE review found no reliable evidence that puberty blockers improve gender dysphoria, mental health, body image, or psychosocial functioning.^11^ The reviewers cautioned that all available studies had ‘very low’ certainty due to biases and confounds. The NICE review of hormone therapy in minors with gender dysphoria identified similar shortcomings, concluding there was very low certainty of benefits that must be weighed against the largely unknown long-term safety profile of these treatments.^10^

After initial support for the GAMOC, international medical authorities have started to remove their endorsements. In 2020, Finland revised its GAMOC guidelines based on a systematic review of the evidence to prioritise psychological interventions over medicine and surgery, particularly for youth with no childhood history of gender dysphoria.^9^ In 2022, the Karolinska Hospital in Sweden, following its own systematic review of the evidence,^8^ issued a new policy statement ending puberty blockers and hormone therapy for minors. Hormone therapy is still allowed after 16 but only in research settings.

Historically, alternative approaches to the treatment of minors presenting with gender dysphoria included watchful waiting^12^ and psychotherapy. Watchful waiting is predicated on high resolution of gender dysphoria after puberty and focuses on the treatment of psychiatric comorbidity and personality pathology.^13^ A representative psychotherapeutic approach to gender dysphoria is Gender Exploratory Psychotherapy (GED),^14^ which considers diverse aetiologies of gender dysphoria, including trauma, internalised homophobia, and comorbid psychiatric conditions such as autism spectrum disorder, borderline personality disorder, and eating disorders. GED provides a supportive approach to clarifying and addressing underlying aetiological factors and dynamics. GED has been erroneously conflated with conversion therapy by critics.^15^

Differing conceptualisations of the clinical phenomena of regret, desistance, and detransition^16,17^ (Table 1) represent another schism between advocates and critics of the GAMOC.^18^ Early studies reporting rare desistance had serious flaws including a narrow definition of desistance and inadequate follow-up. More recent studies reported significant rates of detransition,^19^ but ongoing methodological limitations mean the true rate remains unknown pending well designed longitudinal studies.^20^ These findings, alongside the increasing prominence of detransition stories in the media, and the growing incidence of medical malpractice litigation^21^ all suggest caution over the expansion of the GAMOC in Australian and New Zealand gender services. It seems prudent to consider why so many patients now request life-altering medical and surgical interventions to address issues of identity, and why there has been such a rapid growth of the GAMOC in public health services despite the known and unknown risks, before rapidly increasing the use of largely untested social, medical, and surgical interventions on a vulnerable population. This paper attempts to provide answers using the concepts of abnormal illness behaviour (AIB)^22,23^ and abnormal treatment behaviour (ATB; Table 2).

**Table 2.** Abnormal illness behaviour and abnormal treatment behaviour

Abnormal Illness Behaviour^22,23^
• *Sick role*: an individual affected by illness is granted special treatment as long as they cooperated with others to achieve health as soon as possible • *Abnormal illness behaviour (AIB)*: a persistent maladaptive mode of behaviour by a patient in relation to their health that refuses to accept a competent doctor’s formulation of the illness and the recommended treatment • *Diagnosis of AIB*: *AIB* is diagnosed by a doctor, based on patients’ distortions of the objective facts of their health status associated with complaints and demands • *AIBs and mental illness*: It is uncommon for *AIBs* to involve the affirmation of mental illness, because of the minimal secondary gains available from mental illness due to associated stigma • *Analytic implications of AIB*: ‘It seems reasonable to anticipate that the study of *AIB* syndromes may shed light on societal attitudes to health, illness, and disease, and in particular on the function of the physician in the unusually sensitive nodal point he occupies at the intersection of the psychological, social, and biological systems, which influence the health-illness dimensions of human behaviour’ – Pilowsky, 1978:p136
Pilowsky’s model of AIB^23^
Somatically focused abnormal illness behaviour 1. Illness affirming a. Motivation predominantly conscious b. Motivation predominantly unconscious 2. Illness denying a. Motivation predominantly conscious b. Motivation predominantly unconscious Psychologically focused abnormal illness behaviour 3. Illness affirming a. Motivation predominantly conscious b. Motivation predominantly unconscious 4. Illness denying a. Motivation predominantly conscious b. Motivation predominantly unconscious
Abnormal Treatment Behaviour^24^
• *Assumptions of AIB*: *AIB* assumes a reasonable doctor with a linear cause-effect model of illness shared by patients • *Extension of AIB*: *ATB* assumes a reasonable patient and the possibility of an unreasonable doctor affected by biases, such as the tendency to favour physical over psychological mechanisms, even when physical mechanisms failed to explain all patient experiences • *Abnormal treatment behaviour (ATB)*: a persistent maladaptive mode of behaviour by a doctor in relation to their treatment that refuses to accept a competent patient’s experience of an illness in the context of their life • *Analytic implications of ATB*: Understanding doctor–patient relationships requires an awareness of the bio-psycho-social determinants of the behaviour of both parties
Singh’s model of ATB^24^
Somatically focused abnormal treatment behaviour 1. Illness affirming a. Motivation predominantly conscious b. Motivation predominantly unconscious. 2. Illness denying a. Motivation predominantly conscious b. Motivation predominantly unconscious Psychologically focussed abnormal treatment behaviour 1. Illness affirming a. Motivation predominantly conscious b. Motivation predominantly unconscious 2. Illness denying a. Motivation predominantly conscious b. Motivation predominantly unconscious

## Discussion

It may be anticipated that those who insist that gender diversity does not involve psychopathology will argue that abnormal illness behaviours (AIBs) and abnormal treatment behaviours (ATBs) should not be applied to the GAMOC. However, gender dysphoria remains a DSM-5-TR diagnosis, gender incongruence is an ICD-11 diagnosis within the chapter on sexual disorders and sexual health, and the doctor–patient relationship is a core component of the GAMOC. Thus, AIB and ATB are relevant to the GAMOC whether it is assumed that gender diversity involves psychopathology or not.

Applying the ideas of abnormal illness and abnormal treatment behaviours to the context of gender dysphoria allows the insight that the diagnosis, formulation, and management of patient presentations by doctors can be subverted by unconscious dynamics affecting patients and clinicians. AIB provides a framework for understanding why so many young people now regard lifetime medicalisation as an attractive solution to potentially transitory gender dysphoria, regardless of trauma, internalised homophobia, and other comorbid psychopathologies. ATB provides a framework for understanding why some doctors and health professionals are so committed to the GAMOC despite the limited evidence of benefits, and poorly researched but certainly significant risks of adverse effects and complications, including loss of fertility, loss of sexual function, reduced life expectancy, and regret/desistance/detransition.

One of the great advantages of psychiatry is access to diverse theories expanding on the medical model to reveal the unconscious processes that influence patient and clinician behaviours. This allows for more complete formulation of patient presentations, and a greater range of effective ways of helping them. Table 3 presents features of the fictional case of a patient named Stevie that we will use to demonstrate some ways in which the concepts of AIB and ATB can provide insight into the questions raised above.

**Table 3.** Clinical Case with features of AIB and ATB

The following case is a fictional composite constructed to illustrate features of AIB and ATB. All demographic, clinical, and physical features have been selected so the case does not resemble any actual case of which we are aware
Stevie is a tall, thin 20-year-old single transgender woman born in Sudan and emigrating to Australia as an infant. Her mother died soon after arriving in Australia, and she now lives with long-term supportive foster parents in a large metropolitan centre. Her foster parents have Western European background, and Stevie has social relationships with both her foster parents’ networks and with the local Sudanese community. She receives a Disability Support Pension for intellectual disability. She was admitted to the local hospital with suicidal ideation. No precipitant was identified
DSM-V diagnoses:
• Major depressive disorder with anxiety
• Social anxiety disorder
• Chronic complex PTSD
• Gender dysphoria
• Intellectual disability – mild
Medications at presentation:
• Sertraline 50 mg daily (commenced at admission)
• Spironolactone 100 mg bd
• Estradiol 50 mcg patch
There had been no previous psychiatric medication trials
Stevie demonstrated the following pre-existing vulnerabilities:
1. A history of suicide attempts and self-injury, in the setting of rejection
2. A history of violence and poor impulse control
3. Binge/purge behaviours not meeting diagnosis
4. Estrangement from her family of origin: Her father was murdered in Sudan as part of a genocide, and her mother died of an unknown illness soon after arriving in Australia. She has an older brother who was removed from foster care and placed in youth detention due to violent offences associated with substance abuse, including violence directed against Stevie
5. Perceived abandonment by the Sudanese community
6. Bisexual orientation and sexual function intact
7. Substance (alcohol, tobacco, and marijuana) misuse
8. No employment history
9. Experience of repeated sexual assault by her brother from age six
10. Male body habitus
Features of gender-affirming care:
1. Stevie had never had bone density studies or neuroradiology, and there had been no recent neuropsychological assessment
2. Recent coming out as transgender (age 16), followed by foster family support but Sudanese community rejection of the new identity
3. Uncertainty about her aims and objectives for GAC.
4. She had been seeing a psychologist to address instability in her intimate relationships for 18 months. During the early days of the Covid pandemic and in the context of the painful end of a sexual relationship with a young woman, Stevie described confusion and ambivalence about her gender identity, which at the time was male, consistent with her natal sex. Her psychologist referred her to a psychiatrist known to practice gender-affirming care, and she was diagnosed with gender dysphoria and inducted into the GAMOC under the psychiatrists’ care after a single telehealth interview
5. Stevie had been treated with a puberty blocker for 6 months before commencing estradiol 50 mcg patch (over 12 months) and spironolactone 200 mg (over 12 months). She believed oestrogen may have helped reduce the frequency and intensity of her episodes of anger
6. She had received most of her information about social, medical, and surgical transition from YouTube and was not concerned about side effects or complications of gender medication or surgery

Let us consider fictional patient Stevie through the lenses of AIB and ATB.

The history includes repeated sexual trauma as a young boy at the hands of the brother; manifest in chronic complex PTSD; intellectual disability associated with concrete thinking; disrupted attachment and abandonment, with the early death of her mother and murder of her father; and perceived rejection from her culture as the result of bisexual orientation, pre-existing her current gender confusion and possibly associated with cognitive dissonance and the search for a meaningful identity which is developmentally consistent with her age.

Embracing a new identity affirmed by professionals, foster family, and gender-affirming social networks appears likely to allow Stevie to receive validation and support in a socially accepted way and deny male aspects of herself that she associates with violence, rejection, and death. It may also be satisfying to substitute the validation of the dominant culture for the rejection of the male-dominated Sudanese minority. The expression of gender diversity has provided Stevie with peer support often difficult for children with intellectual disability to access. On the other hand, this new identity and its powerfully reinforcing social contingencies might distract Stevie from conscious awareness of the underlying causes of her distress and therefore perpetuate her depression and anxiety by focussing on the relief of symptoms rather than the treatment of pathology.

The competent interpretation of such psychodynamic processes makes unquestioning affirmation impossible, because it must consider whether the self-reported identity is mistaken, misleading, or frankly factitious. For example, an exploration of the possibility that a gender identity has been reported for the purposes of secondary gain would violate the principle of unquestioning affirmation.

Among the ATBs expected due to uncritical gender affirmation, the most serious are a great reduction in the likelihood that clinicians will robustly assess, diagnose, and treat psychopathological factors that may contribute to gender dysphoria. For example, in Stevie’s case, the inability to explore her reported gender identity would prevent exploration of the psychic effects of her early abuse by her brother and the association between maleness, violence, and the loss of control.

It is useful to consider how Stevie’s case fits into Singh’s nosological framework for ATBs (Table 2).^24^ The initiation of puberty blockers and hormone therapy instead of a psychotherapeutic approach such as Gender Exploratory Psychotherapy suggests the possibility of somatically focussed, illness affirming ATB. The question whether the psychiatrist’s motivation is conscious, unconscious, or a mix of both is a complicated one that depends upon their level of self-awareness.

It is instructive to consider how the different diagnostic approaches taken in the ICD-11 and DSM-5-TR might influence the probability of ATBs in Stevie’s treatment. While the DSM-5-TR still considers gender dysphoria to be a mental illness, the ICD-11 treats gender incongruence as a normal feature of human development. Thus, the diagnosis of gender incongruence could redirect affirming clinicians away from the psychopathological factors outlined above leading to under-diagnosis of Stevie’s depression, anxiety, and failure to recognise mechanisms such as compromised identity formation. This focus on gender incongruence to the exclusion of other causes of distress is called diagnostic overshadowing and can have undesirable effects including under-referring (e.g. for trauma focussed psychotherapy and exploratory psychotherapy), under-treating (with delay of and then inadequate trial of antidepressant medication), and consequent prolongation of illness behaviour. Diagnostic overshadowing was one of the serious problems with the GAMOC revealed by the Cass Review which led to the suspension of the gender service at the Tavistock Institute in the United Kingdom.^21^

In addition, it may be argued that if medical affirmation becomes the primary treatment modality, that there is overtreating of the gender identity (with consequent risks of metabolic disorder and subsequent cardiovascular disease), while relatively ignoring other significant diagnoses and dynamics. In Stevie’s case, there had been no bone density or neurological radiology and no recent neuropsychological update, perhaps in keeping with under-investigation associated with ATB.

Additionally, this case could be argued as an example of psychologically focussed ATB, illness denying, motivation conscious (collusion with patient) and motivation unconscious (countertransference, judgemental attitude towards psychiatry, and identification with patients with an overinclusive view of normality). In the GAMOC, the patient is idealised as the unquestionable gender identity expert, and the clinician has the subordinate role of uncritical affirmation to facilitate social, medical, and surgical support. As a form of unconscious collusion, this encourages medical and surgical over-treatment,^22^ under-treatment of psychiatric disorders, under-investigation of other factors, and may prolong illness over and above the probability of the lifelong trajectory of affirmation interventions. Whatever the causes, it is certainly true that there have been exponential increases in gender-affirming interventions in Australian and New Zealand minors, including puberty blockers and hormone therapy, since 2010.^22^

## Conclusion

This paper explores abnormal illness behaviours (AIBs) and abnormal treatment behaviours (ATBs) that may arise in the context of the gender-affirming model of care (GAMOC) by reinforcing the expression of gender diverse identities, overshadowing differential diagnoses, and redirecting clinician attention away from psychopathological contributions to gender diversity. This paper does not propose that AIB and ATB are exclusive or even preferred models of gender diversity, but merely that they contribute to understanding such patients and improving their care.

Pilowsky and Singh emphasise the impact of social, political, and cultural forces upon the doctor–patient relationship.^23–25^ Singh acknowledges that economic factors influence diagnostic and prescribing patterns, as well as patient’s families. Adopting the GAMOC may have significant potential financial and career consequences, including industry sponsorship, academic advancement for those who subscribe to progressive politics, and public recognition.

When Pilowsky and Singh wrote their articles, mainstream medical ethics balanced the principles of autonomy, non-maleficence, and beneficence. It can be argued that the rise of the GAMOC over the last 10 years mirrors the growing dominance of the principle of autonomy over the broader society. Over the same period, the GAMOC has been imbued with the status of a human rights issue, reflecting the significant influence of political and social movements over patients and physicians. However, while we acknowledge that adults can choose to prioritise autonomy over safety by exercising human rights that might include risky behaviours, we believe that doctors continue to have an ethical responsibility to balance autonomy and safety on behalf of their patients. This is particularly true for minors, who have widely varying ability to balance autonomy, safety, and other goods.

In our opinion, the uncritical affirmation by clinicians of the self-reported gender identity of all patients irrespective of their history, personality, comorbidities, and circumstances carries a high risk of concealing the unconscious motivations of patients and clinicians alike. If the medical profession does not urgently address this problem, it will be responsible for any harms that result, and risk loss of public confidence.
